# Essential Oils From the Flowers of *Callistemon viminalis* (Myrtaceae): Circadian Variation, Chemical Profile, and Potential in the Control of Phytopathogens

**DOI:** 10.1002/cbdv.71922

**Published:** 2026-11-14

**Authors:** Chrystiaine Helena Campos de Matos, Paloma Carolina de Lima Carvalho, João Paulo de Sousa, Luís Augusto Calsavara Costa, Jesus David Sierra Martinez, Alex Oliveira Botelho, Moacir Rossi Forim, Gracielle Oliveira Sabbag Cunha, Andreia Pereira Matos, Cássia Cristina Fernandes Alves, Cristiane de Melo Cazal

**Affiliations:** ^1^ Goiano Federal Institute of Education, Science and Technology, Campus Rio Verde Rio Verde Goiás Brazil; ^2^ Federal Institute of Education, Science and Technology of Southeastern Minas Gerais, Campus Barbacena Barbacena Minas Gerais Brazil; ^3^ Department of Chemistry, Natural Products Laboratory Federal University of São Carlos São Carlos São Paulo Brazil; ^4^ Federal Institute of Education, Science and Technology of Goiás, Campus Anápolis Anápolis Goiás Brazil

**Keywords:** *Botrytis cinerea*, circadian cycle, *Colletotrichum gloeosporioides*, eucalyptol, fungicide

## Abstract

This study aimed to evaluate the influence of the circadian cycle on the chemical composition and yield of essential oils (EOs) from *Callistemon viminalis* flowers, as well as to assess their biological activity against the phytopathogenic fungi *Botrytis cinerea* and *Colletotrichum gloeosporioides*. EOs were obtained from fresh flowers collected at 12 a.m., 6 a.m., 12 p.m., and 6 p.m. of the same day and analyzed by gas chromatography coupled to mass spectrometry (GC‐MS). The in vitro antifungal assay was performed using the disk diffusion method. The EO of *C. viminalis* showed yields between 0.32% and 1.84%. The major constituents of the EOs were eucalyptol, α‐pinene, and α‐terpineol for samples collected at 12 a.m., 6 a.m., and 12 p.m., and eucalyptol and α‐pinene for flowers collected at 6 p.m. The EO of *C. viminalis* proved effective in reducing the mycelial growth of *C. gloeosporioides* and *B. cinerea* at 500 µL.mL^−1^

## Graphical abstract

The circadian cycle influences the yield and chemical composition of essential oils from Callistemon viminalis flowers. Eucalyptol and α‐pinene are identified as major constituents throughout the evaluated periods. The essential oils exhibit antifungal activity, reducing the mycelial growth of Colletotrichum gloeosporioides and Botrytis cinerea at 500 µL mL^−^^1^.

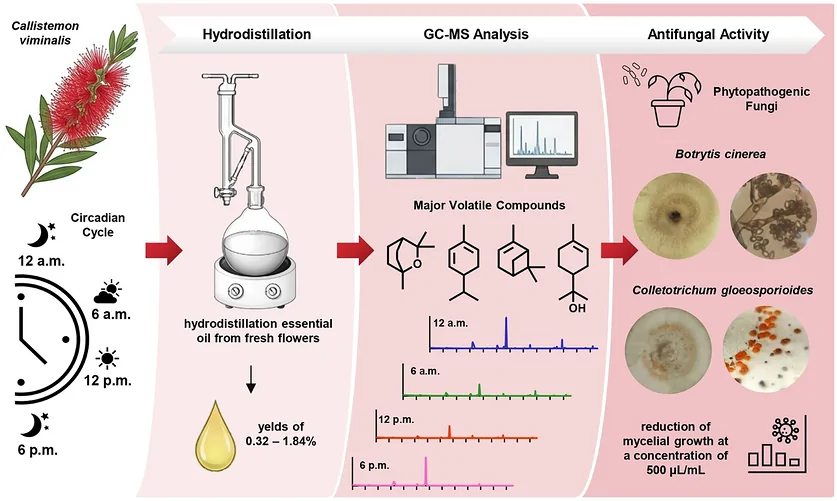

## 1 Introduction

The Myrtaceae family is one of the largest in the plant kingdom, considered the eighth largest family of flowering plants. It includes species ranging from fruit trees to woody shrubs and occurs in countries with tropical and subtropical climates around the world, in addition to having ecological and economic importance [1]. The essential oils (EOs) of various species in the Myrtaceae family exhibit active biological properties, demonstrating the importance of this family as a potential source of substances for technological application [2]. Flowers are an important source of plant and spice EOs and can be used as repellents for mosquitoes, insects, and others. Secondary metabolites isolated from flowers, such as flavonoids, terpenoids, alkaloids, phenylpropanoids, and organic acids, exhibit diverse biological effects [3].

EOs have shown potential for use as an alternative for pathogen control [4]. Since fungal diseases affect the productivity and quality of food products, the control of phytopathogens in agricultural production is of global interest, as they cause significant economic losses to producers [5]. Common pathogenic fungal infestations include gray mold (*Botrytis cinerea*), white rot (*Coniothyrium diplodilla*), anthracnose (*Colletotrichum gloeosporioides*), and others [6].

The genus *Callistemon*, belonging to the Myrtaceae family, represents a group of shrubs and small trees known for their richness in EOs, with reported fungicidal [7], antimicrobial [8], antioxidant [8, 9], and insecticidal [10] activities. The EOs from species of the *Callistemon* genus predominantly contain substances from the terpene class, including the monoterpenes eucalyptol, α‐pinene and α‐terpineol [11]. However, the chemical composition and concentration of the major compounds can vary depending on geographic location, environmental conditions, and the plant's circadian cycle [12].

A great variability in the chemical components of EOs, attributed to several factors such as climatic, seasonal, geographical and circadian conditions, harvest period, storage conditions and distillation technique, can be observed [13]. As a consequence, the biological properties of EOs, which are directly related to their chemical compositions, can also be affected by these variations [14]. For this reason, the study of the chemical variability of EOs is of extreme scientific importance, aiming to better understand the special metabolism of the species. Thus, the present study aimed to evaluate the influence of the circadian cycle on the chemical composition and yield of EOs from *Callistemon viminalis* flowers and to assess their biological activity against the phytopathogenic fungi *Botrytis cienrea* and *Colletotrichum gloeosporioides*.

## 2 Results and Discussion

### 2.1 Study of the Circadian Cycle—EO Content

The EO from *C. viminalis* flowers presented a yellowish color and a peculiar, pleasant aroma. The content varied between 0.32% and 1.84% (Figure 1) for flowers collected at different times of day. For flowers collected at 12 a.m., a yield of 0.93% was achieved. For the extraction of flowers collected at 6 a.m., a yield of 1.84% was obtained. For the sample collected at 12 p.m, a content of 1.14% was obtained, and for the flowers collected at 6 p.m., a yield of 0.32% was achieved. Despite the variations, the samples collected at 12 a.m. and 12 p.m. did not differ statistically in terms of their yield values.

**FIGURE 1:**
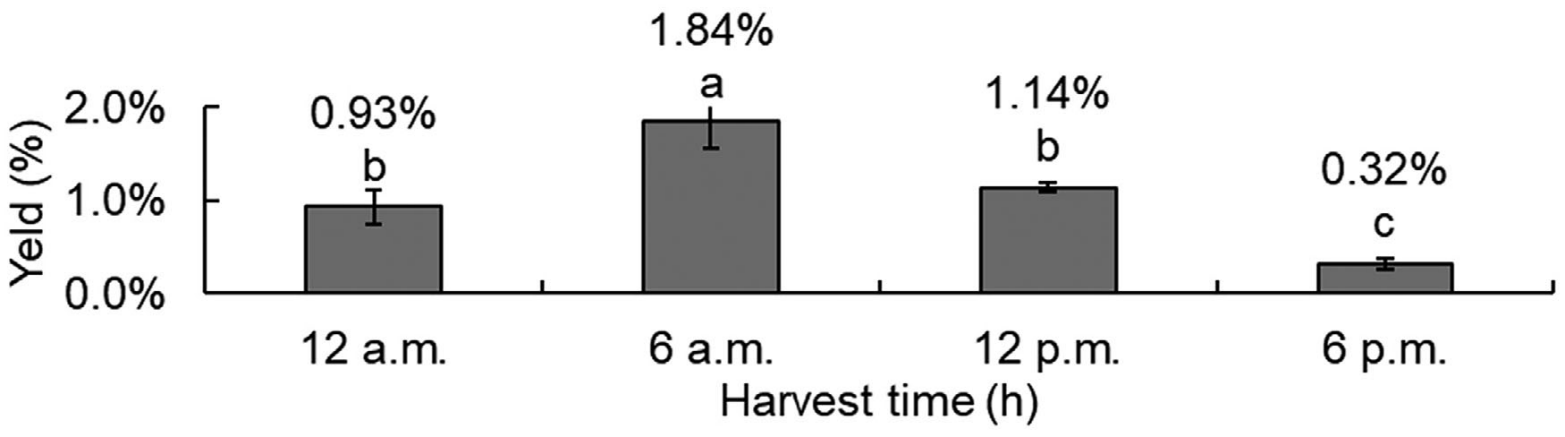
Essential oil content of *Callistemon viminalis* flowers collected at different times of day. *Means followed by the same letter do not differ significantly by Tukey's test at 5% probability.

Species of the genus *Callistemon* have already been investigated for volatile oil content and composition. There are few reports in the literature on the yield of *C. viminalis* EO. Pires et al. [15]. observed a yield of 0.30% for the EO of *C. viminalis* fresh flowers collected in Brazil; however, in this study, the time of collection of the plant material was not defined. Other species of the same genus have shown different EO contents. The EOs of *Callistemon citrinus* flowers have shown a content of 0.80% for plants collected in South Africa [16] and 0.12% in the Western Himalayas [17]. The flowers of *Callistemon lanceolatus* and *Callistemon polandii* yielded 0.2% and 0.1%, respectively [18]. The observed yield for the EO of *Callsitemon viridiflorus* flowers was 1.5% [19]. These variations in EO yield may be related to the environmental conditions of the plant's location. In the present study, the variation in temperature and relative humidity was proportional to the increase in content, and the EO of *C. viminalis* flowers had its highest yield when the humidity index was 93%, the highest value for the day and at the lowest temperature (14.5°C), as can be seen in Table 1.

**TABLE 1:** Relationship between the climatic conditions of the city of Barbacena—MG on the day of collection of *Callistemon viminalis* flowers and the essential oil content.

Time	Precipitation (mm)	Humidity (%)	Temperature (°C)	EO content (%)•
12 a.m.	0	73	19.2	0.93^b^ ± 0.18
6 a.m.	0	93	14.5	1.84^a^ ± 0.28
12 p.m.	0	76	18.3	1.14^b^ ± 0.05
6 p.m.	0	34	28.4	0.32^c^ ± 0.05

^*^Means followed by the same letter do not differ significantly by Tukey's test at 5% probability.

*Source*: Instituto Nacional De Meteorologia (INMET) [20].

Santos and colleagues [21] evaluated the seasonal variability in the yield of EO from *Psidium friedrichsthalianum*, a plant belonging to the Myrtaceae family. A variation in the average EO yield was observed in the dry season (August to February) compared to the rainy season (March to May). There was significant seasonal variability in relation to sunshine and humidity. However, no variation was observed in relation to temperature. Data that differ from those observed for *C. viminalis*.

### 2.2 Chemical Composition of EOs

Chemical analysis of the EOs revealed 17, 23, 29, and 20 chemical compounds for the samples at 12 a.m., 6 a.m., 12 p.m., and 6 p.m., respectively. In general, the major compounds of the EOs did not change throughout the day. All samples showed the presence of eucalyptol, α‐pinene, α‐phellandrene, linalool, and α‐terpineol in significantly higher quantities than the other substances. However, the relative levels of each compound changed throughout the circadian cycle. The relationship of the major constituents throughout the day can be seen in Figure 2. A variation in the chemical compounds of *C. viminalis* EO profiles was observed depending on the different collection times. The results showed that eucalyptol was the main chemical marker. Other prominent compounds include α‐pinene, α‐terpineol, α‐phellandrene, and linalool (Figure 2 and Table 2). There are few studies that report the composition of the EO of *C. viminalis* flowers. Zhang and colleagues (2025) [9] analyzed the components present in the EO of *C. viminalis* flowers. The major compound was eucalyptol (54.21%), followed by α‐terpineol (96.02%) and β‐caryophyllene (3.04%). Previously, the EO of *C. viminalis* flowers was studied by Pires et al. (2013) [15] and it was observed that the major components were: eucalyptol (42.39%), tricyclene (28.08%), α‐terpineol (6.67%), α‐phellandrene (6.64%) and limonene (5.46%).

**FIGURE 2:**
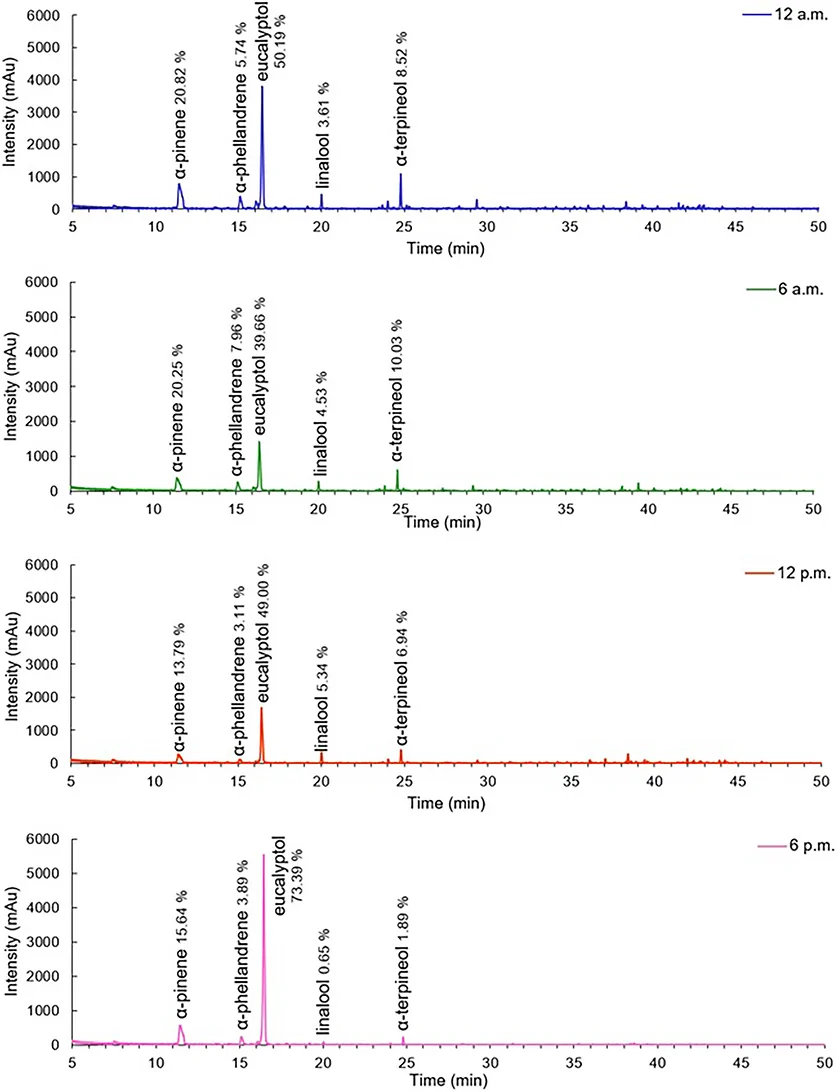
Chromatograms showing the relative content of the major compounds in the essential oils of *Callistemon viminalis* flowers collected at different times of day.

**TABLE 2:** Chemical composition of the essential oil from *Callistemon viminalis* flowers at different collection times.

Compound	Content (%)				RT (min)	RI exp^a^	RI lit^b^
	12 a.m.	6 a.m.	12 p.m.	6 p.m.			
4‐hydroxy‐4‐methyl‐2‐pentanone	1.51	3.69	3.49	1.51	7.5	843	831
α‐pinene	20.82	20.25	13.79	15.64	11.4	933	932
β‐pinene	—	0.56	0.5	0.27	13.6	976	974
myrcene	—	0.65	0.43	0.26	14.4	992	988
α‐phelladrene	5.74	7.96	3.11	3.89	15.1	1007	1002
ρ‐cymene	2.6	2.2	1.12	0.78	16.1	1025	1020
eucalyptol	50.19	39.66	49	73.39	16.4	1032	1026
*trans*‐ocimene	—	0.38	—	0.13	17.3	1048	1044
γ‐terpinene	0.6	0.54	0.24	0.24	17.8	1058	1054
terpinolene	0.56	0.6	0.29	0.3	19.2	1085	1086
linalool	3.61	4.53	5.34	0.65	20.0	1101	1095
terpinen‐4‐ol	1.51	2.09	1.81	0.2	24.0	1180	1174
α‐terpineol	8.52	10.03	6.94	1.89	24.8	1195	1186
geraniol	—	0.87	0.24	—	27.6	1251	1249
eugenol	—	—	0.15	—	32.3	1351	1356
α‐copaene	—	—	0.17	—	33.4	1374	1374
isoledene	—	—	0.21	—	33.1	1369	1374
geranyl acetate	0.27	0.43	0.28	—	33.5	1378	1379
β‐elemene	—	—	0.13	—	34.0	1388	1389
α‐gurjunene	0.17	0.16	0.44	—	34.7	1405	1409
β‐caryophillene	—	—	—	0.14	35.3	1417	1417
aromadendrene	0.53	0.86	1.45	0.07	36.1	1436	1439
allo‐aromadendrene	—	0.77	2.04	0.07	37.1	1458	1458
γ‐gurjunene	—	—	0.27	—	37.6	1471	1475
cis‐β‐guaiene	—	—	0.14	—	38.3	1486	1492
viridiflorene	—	2.04	4.32	0.13	38.4	1489	1496
valencene	1.63	—	—	—	38.4	1489	1496
bicyclogermacrene	—	0.27	0.67	0.27	38.6	1493	1500
δ‐cadinene	0.19	—	0.64	0.03	39.6	1516	1522
sphatulenol	1.02	1.07	2.22	0.14	42.0	1571	1577
globulol	—	0.2	—	—	42.7	1587	1590
guaiol	0.53	0.19	0.41	—	42.8	1589	1600
rosifoliol	—	—	0.16	—	43.2	1599	1600

**RT (min)**: representative retention time obtained for each compound in a sample in which it was identified.

**RI exp^a^**: experimental retention index calculated relative to n‐alkanes (C7–C40).

**RI lit^b^**: retention index reported in the literature [27].

The predominance of eucalyptol in samples collected during the day, particularly at 6 p.m., suggests that its biosynthesis may be modulated by environmental factors such as temperature and solar radiation intensity. This pattern is consistent with evidence that exposure to radiation, especially under conditions of higher light intensity, can induce changes in the secondary metabolism of plants, promoting increased production of monoterpenes and other bioactive compounds associated with protection against abiotic stresses (Crestani et al., 2024) [22]. In this context, eucalyptol can be interpreted as a potential metabolic marker of the physiological response of *C. viminalis* to environmental variations in light and temperature throughout the day, as reported by the following authors [23, 24, 25, 26].

All constituents present in the EO samples of *C. viminalis* flowers can be found at different times and are presented in Table 2.

Figure 3 presents the results of the hierarchical heatmap analysis of the circadian variation of the EOs. The data revealed clustering patterns based on the relative levels of the compounds throughout the circadian cycle. The chromatic arrangement (gradient from blue to red) highlights striking differences in chemical composition between the collection times.

**FIGURE 3:**
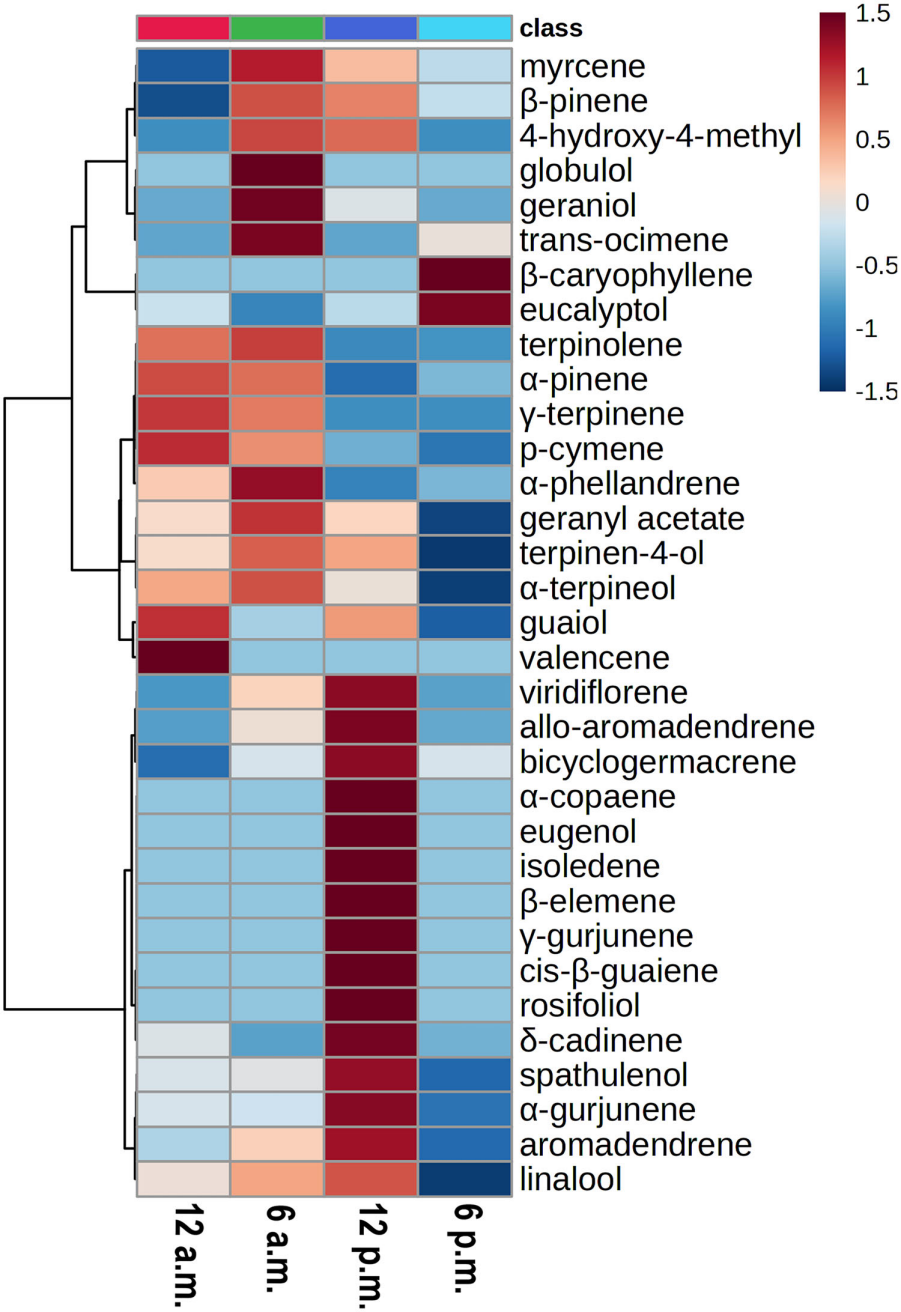
Heatmap analysis for the different chemical constituents of *Callistemon viminalis* flower essential oils throughout the circadian cycle.

The hierarchical heatmap (Figure 3) revealed clustering patterns based on the relative levels of compounds throughout the circadian cycle. The chromatic arrangement highlights striking differences in chemical composition between collection times. Eucalyptol, the main constituent in all samples, reached its highest relative concentration at 6 p.m. (73.39%), which can be explained by the strong influence of light and temperature on monoterpene biosynthesis, as previously reported by Hashemifar et al. [28] and Yu et al. [29] in transcriptomic analyses of aromatic plants. Among other monoterpenes, linalool peaked at 12 p.m. (5.34%), α‐terpineol at 6 a.m. (10.03%), and p‐cymene at 12 p.m. (2.60%), a reduction in specific temporal regulation of the compound within the same biosynthetic class.

Terpinolene, geranyl acetate, globulol, and geraniol are found in higher concentrations at 6 a.m. indicating endogenous regulation associated with the transition from the nocturnal to the diurnal cycle. Valencene was detected only at 12 a.m. (1.63%), while globol was found only at 6 a.m. (0.20%), showing an accumulation of these sesquiterpenes during a specific period. β‐caryophyllene was detected exclusively at 6 p.m. (0.14%), along with the highest concentration of eucalyptol, differentiating the 6 p.m. profile from all others. Several compounds, such as eugenol, α‐copaene, isoledene, β‐elemene, γ‐gurjunene, cis‐β‐guaiene, and rosifoliol, were detected exclusively at 12 p.m., indicating the activation of specific sesquiterpene biosynthetic pathways during peak daylight hours, localized to defense responses against herbivores and pathogens [30].

The results found at all four time points included 4‐hydroxy‐4‐methyl‐2‐pentanone, α‐pinene, α‐phellandrene, p‐cymene, eucalyptol, γ‐terpinene, terpinolene, linalool, terpinen‐4‐ol, α‐terpineol, aromadendrene, and spathulenol, proportioning constitutive production throughout the circadian cycle. The differential accumulation of these compounds, independent of the time of collection, demonstrates that multiple biosynthetic pathways of monoterpenes and sesquiterpenes are modulated by the circadian rhythm, which is consistent with transcriptomic studies that point to the influence of biological clock regulatory genes on the expression of enzymes in the mevalonate pathway and the MEP/DOXP pathway [31, 32]. Taken together, these results demonstrate that the timing of collection is a critical experimental variable for the EO of *C. viminalis*, with direct implications for its chemical profile and potential biological activity, including applications in the control of phytopathogens.

Recently, Silva and colleagues [26] analyzed the volatile constituents of EOs from fresh *C. viminalis* flowers by GC‐MS and GC‐FID. The main constituent was eucalyptol (1,8‐cineole) (62.3%), followed by spathulenol (10.8%), alpha‐pinene (10.7%), and viridiflorol. In a previous work, Pires and colleagues [15] analyzed the EO of *C. viminalis* flowers and obtained eucalyptol (42.39%) as the major constituent, in addition to tricyclene (28.8%) and α‐terpineol (6.67%). Furthermore, the monoterpenes α‐phellandrene (6.64%) and limonene (5.46%) were also identified. Both plants were collected in Goiás, a state in Brazil.

The association between the results of the VIP scores (PLS‐DA) and the hierarchical heatmap confirms the existence of marked circadian chemical variations in the EOs of *C. viminalis*. The precise determination of key compounds and their peak production times provides a solid scientific basis for choosing harvesting and processing strategies aimed at increasing the effectiveness of EO applications, especially in the context of phytopathogen biocontrol.

### 2.3 Study of the Biological Activity of EO

#### 2.3.1 Content and Chemical Composition of the EO

The EO of *C. viminalis* flowers collected at 6 p.m. at Location 2 showed a yield of 0.73%. Chemical analysis revealed 36 chemical compounds, the major ones being: eucalyptol (67.06%), α‐pinene (11.70%), α‐phellandrene (4.88%), α‐terpineol (3.25%), limonene (3.14%), o‐cymene (1.27%), linalool (1.16%), and terpinen‐4‐ol (1.14%). All constituents present in the EO of *C. viminalis* flowers can be observed in Table 3.

**TABLE 3:** Chemical composition of the essential oil from *Callistemon viminalis* flowers collected at 6 p.m. at Location 2.

Compound	Content (%)	RT (min)	RI exp^a^	RI lit^b^
Hexanal	0.04	5.814	801.6361	801
*trans*‐2‐hexenal	0.44	7.746	849.529	846
*cis*‐3‐hexenol	0.15	7.851	852.1319	850
isopentyl acetate	0.12	8.781	875.1859	869
isobutyl isobutirate	0.09	10.488	914.171	908
α‐tujene	0.46	10.925	922.9426	924
α‐pinene	11.7	11.241	929.2854	932
sabinene	0.02	13.222	969.0486	969
β‐pinene	0.65	13.417	972.9627	974
myrcene	0.96	14.168	988.0369	988
pseudolimonene	0.02	14.801	1000.713	1003
α‐phellandrene	4.88	14.939	1003.374	1002
isopentyl isoburate	0.16	15.41	1012.457	1007
α‐terpinene	0.15	15.498	1014.153	1014
o‐cimene	1.27	15.912	1022.137	1022
limonene	3.14	16.164	1026.996	1024
eucalyptol	67.06	16.308	1029.772	1026
*cis*‐ocimene	0.05	16.606	1035.519	1032
*trans*‐ocimene	0.26	17.118	1045.391	1044
γ‐terpinene	0.53	17.653	1055.708	1054
terpinolene	0.39	19.04	1082.453	1086
linalool	1.16	19.875	1098.554	1095
*cis*‐ρ‐ment‐2‐en‐1‐ol	0.08	21.061	1121.75	1118
borneol	0.08	23.592	1171.3	1165
terpinen‐4‐ol	1.14	23.913	1177.584	1174
α‐terpineol	3.25	24.676	1192.522	1186
α‐gurjunene	0.05	34,664	1402.847	1409
aromadendreno	0.11	36.033	1434.275	1439
allo‐aromadendrene	0.18	36.976	1455.923	1458
viridiflorene	0.39	38.335	1487.121	1496
bicyclogermacrene	0.31	38.527	1491.529	1500
δ‐cadinene	0.05	39.51	1514.079	1522
sphatulenol	0.36	42.124	1574.02	1577
viridiflorol	0.14	42.509	1582.848	1592
globulol	0.1	42.856	1590.805	1590
guaiol	0.06	42.957	1593.121	1600

**RT (min)**: retention time; **RI exp^a^**: experimental retention index calculated relative to n‐alkanes (C7–C40); **RI lit^b^**: retention index reported in the literature [27].

The EO extracted for the antifungal activity evaluation showed variations in total chemical constituents when compared to the EOs extracted for the circadian cycle evaluation of *C. viminalis*. This occurrence can be explained because the production of secondary metabolites is influenced by biotic and abiotic factors that affect the biosynthetic pathway [33, 34]. Therefore, the chemical composition of EOs from the same plant species can vary due to factors such as developmental stages, seasonal variation, cultivation, and biotic and abiotic stress. Climate, temperature, precipitation, day length, photoperiod, nutrients, and hormones all play a role [35].

#### 2.3.2 Antifungal Activity of *Callistemon viminalis* EOs

The EO from *C. viminalis* flowers, regardless of the concentration used, did not show any variation in the percentage of inhibition of mycelial growth (PICM) for *Botrytis cinerea* (Figure 4). Few studies have investigated the antifungal activity of the EO obtained from the flowers of *C. viminalis*. Silva et al. (2024) [26] evaluated the antifungal activity of EOs extracted from the flowers and leaves of this species against *Malassezia furfur*, reporting minimum inhibitory concentrations (MICs) of 62.5 and 50 µg.mL^−^^1^, respectively. Previous studies have also demonstrated that the EO obtained from fresh leaves of *C. viminalis* exhibits antifungal activity against different fungal species, including *B. cinerea* [14], with a MIC of 758 mg.L^−^^1^. Furthermore, Badawy and Abdelgaleil (2014) [36] reported that EOs extracted from *C. viminalis* leaves collected at 6:00 a.m. and 6:00 p.m. achieved 100% inhibition of *B. cinerea* mycelial growth at a concentration of 500 µL.mL^−^^1^. These findings indicate a stronger antifungal activity than that observed in the present study.

**FIGURE 4:**
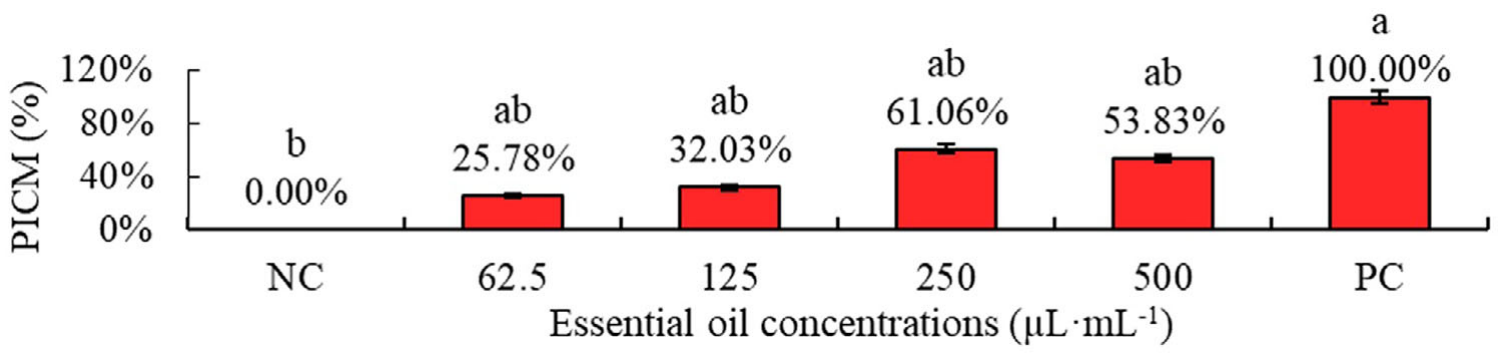
Percentage of mycelial growth inhibition (PICM) of *Botrytis cinerea* in treatments with essential oil from *Callistemon viminalis* flowers collected at 6 PM. *Means followed by the same letter do not differ significantly by Tukey's test at 5% probability. NC: negative control; PC: positive control.

For *C. gloeosporioides*, the EO from *C. viminalis* flowers at 500 µL.mL^−1^ demonstrated potential to control the phytopathogen with a PICM of 57.90% (Figure 5). Matos and collaborators [14] evaluated the EOs extracted from *C. viminalis* leaves against *C. gloeosporioides*; the inhibition of mycelial growth at 500 µL.mL^−1^ was 52.3% and 55.9% for the EOs collected at 6 a.m. and 12 p.m., respectively. EOs extracted from *C. viminalis* leaves were obtained by different methods. The best results were obtained for the NHM EO (natural heating method), which presented MICs of 0.1 µL.mL^−1^ and 0.15 µL.mL^−1^ against *Colletotrichum fructicola* and *Colletotrichum acutatum*, respectively, and MFCs (minimum fungicidal concentration) of 0.75 µL.mL^−1^ and 0.50 µL.mL^−1^ [9].

**FIGURE 5:**
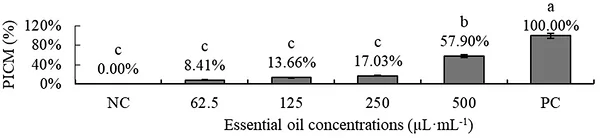
Percentage of mycelial growth inhibition (PICM) of *Colletotrichum gloeosporioides* in essential oil treatments of *Callistemon viminalis* flowers collected at 6 PM. *Means followed by the same letter do not differ significantly by Tukey's test at 5% probability. NC: negative control; PC: positive control.

Antifungal activity has also been reported for EOs from other plant genera containing compounds identified in *C. viminalis* flower EO. The EO of *Alpinia zerumbet* var. *exelsa*, whose major constituents included terpinen‐4‐ol and eucalyptol (1,8‐cineole), exhibited antifungal activity against *Aspergillus brasiliensis*, with terpinen‐4‐ol suggested as an important contributor to this activity [37]. Similarly, rosemary (*Rosmarinus officinalis*) EO, rich in eucalyptol and α‐pinene, as well as these two isolated constituents, showed antifungal activity against *Candida albicans* [38]. These reports further support the antifungal potential of EOs containing monoterpenes also identified in *C. viminalis* flower EO.

The antifungal effect of *C. viminalis* flower EO may be associated, at least in part, with the relative contents of terpinen‐4‐ol, eucalyptol, and α‐pinene. Terpinen‐4‐ol and eucalyptol have been shown to interact with ergosterol monolayers used as model membrane systems, substantially modifying the condensation, morphology, and molecular organization of sterol films, suggesting that their antifungal effects may be associated with alterations in fungal membrane organization [39]. In *Botrytis cinerea*, terpinen‐4‐ol caused alterations in mycelial morphology and cellular ultrastructure, increased membrane permeability, and reduced ergosterol content, indicating the fungal cell membrane as an important target of this compound. Eucalyptol, in contrast, caused serious intracellular damage but only slightly affected *B. cinérea* otherwise. When terpinen‐4‐ol and eucalyptol were used together, their synergistic antifungal activity was significantly higher than that of either component alone [40]. α‐Pinene may also affect fungal membrane function; studies with *Candida albicans* suggest that its fungicidal activity involves interaction with ergosterol in the cell membrane [41]. Thus, the antifungal activity observed for *C. viminalis* flower EO may involve different mechanisms of action of its major constituents, including effects on fungal membranes and intracellular structures.

## 3 Conclusions

It is concluded that the yield of EOs from *C. viminalis* flowers is influenced by the time of collection, suggesting the best yield at collection times with higher humidity levels. In general, the major constituents of the EOs from *C. viminalis* flowers were constant, with eucalyptol, α‐pinene, α‐phellandrene, linalool, and α‐terpineol detected throughout the circadian cycle, and eucalyptol, α‐pinene, α‐phellandrene, α‐terpineol, limonene, o‐cymene, linalool, and terpinen‐4‐ol for the EOs of flowers collected for antifungal activity evaluation. Additionally, the EO of the flower is effective in reducing the mycelial growth of *C. gloeosporioides* and *B. cinerea*, showing better performance at a concentration of 500 µL.mL^−^^1^, with inhibition percentages of 53.83% and 57.9%, respectively. Further studies should be conducted at higher concentrations of EO to achieve statistically equal values to the commercial antifungal, but the results obtained here are promising.

## 4 Experimental Section

### 4.1 Plant Sample and EO Isolation

For the circadian cycle study, *C. viminalis* flowers were collected at different times: 12 a.m., 6 a.m., 12 p.m., and 6 p.m., at the end of winter in September 2023, at Location 1 (Latitude: 21°13'38.5“S; Longitude: 43°45'50.1”W). For the antifungal activity study, *C. viminalis* flowers were collected in autumn at 6 p.m. in May 2022, at Location 2 (Latitude: 21°13'13.3“S; Longitude: 43°46'49.5”W). Both collections were carried out in the city of Barbacena–Minas Gerais, Brazil. The species was identified by Prof. Dr. Leonardo Maurici Borges from the Department of Botany, Federal University of São Carlos (UFSCar). Voucher specimens from each location (C.H.C. de Matos 1 and C.H.C. de Matos 2, for Locations 1 and 2, respectively) were deposited in the Herbarium of UFSCar (SPSC), São Carlos campus, under accession numbers SPSC 9240 and SPSC 9241, respectively.

The flowers were crushed in a blender, and a 200 g sample of plant material was submerged in 1000 mL of distilled water. Subsequently, the samples were subjected to hydrodistillation in a Clevenger‐type apparatus adapted to a 3 L round‐bottom flask, with heating maintained at the minimum temperature necessary for boiling. EO extraction was performed by steam distillation for 1 h. The entire extraction procedure was carried out independently for each biological replicate, totaling three independent hydrodistillations and the oil was extracted for one hour using the steam distillation method. After the extraction process, the hydrosol was subjected to liquid‐liquid partition using 30 mL of dichloromethane, in triplicate, totaling 90 mL of solvent. The non‐polar phase was transferred to a flat‐bottom flask. Anhydrous magnesium sulfate was used as a drying agent, which was subsequently removed from the sample by simple filtration. Dichloromethane was then removed under reduced pressure using a rotary evaporator. After liquid–liquid partition, the remaining hydrosol was discarded. Immediately after complete solvent evaporation, the EO was weighed on an analytical balance to determine its mass and stored at 4°C until further analysis. After obtaining the EO and performing mass measurements on an analytical balance, the content was calculated using Equation 1.

(1) $$\mathrm{Yield}\;\%\;=\frac{mass\;of\;essential\;oil\;\left(g\right)}{mass\;of\;plant\;material\;\left(g\right)}\;\times \;100\%$$

The results of the OE performance were performed in triplicate and analyzed by comparing the mean and standard deviation using the multivariate analysis method ANOVA, with Tukey's post‐hoc test at a 5% probability level using the SISVAR computer program (Version 5.8) [42].

### 4.2 GC–MS Analysis

The EO obtained were analyzed by gas chromatograph‐mass spectrometry (GC‐MS). For chemical characterization, a representative sample prepared by pooling the three biological replicates was analyzed by GC–MS, and no additional instrumental replicates were performed. Samples were processed via a Combi PAL AOC‐5000 autoinjector, Restek Rtx‐4 ms fused silica capillary column (30 m × 0.25 mm × 0.25 µm), in a sequential mass spectrometer (MSTQ8030, Shimadzu), and an ionization detector of electron impact (IE) (70 eV). The temperature profiles were 40°C for 3 min, rising to 150°C (at 3°C min^−1^), and then 280°C (at 8°C min^−1^) remaining for 5 min. The injector and detector temperature were 250 and 280°C, respectively. The carrier gas was helium with injection pressure of 58.6 KPa and flow rate of 34 mL min^−1^. Identification of the components of the EOs was based on the linear retention index (Kovats index—KI) related to retention times of a homologous series of n‐alkanes (C7 to C40) and in the fragmentation pattern observed in the mass spectra, comparing with the GC‐MS system libraries (Nist 05, Nist 08, Nist 11 and Wiley 7v100) and with the literature spectra [27].

The principal component analysis (PCA) was applied to verify the interrelation in the composition of the EOs extracted at different times and collected at different times (PAST, version 4.03) [43]. Multivariate statistical analysis was performed using the MetaboAnalyst 6.0 platform. Data were subjected to the autoscaling standardization method (centered on the mean and scaled by the standard deviation) to equalize the contribution of major and minor compounds. A heatmap was generated using Pearson's distance as a measure of dissimilarity and Ward's clustering method for hierarchical clustering of compounds, allowing visualization of co‐variation patterns in the EO composition over the evaluated period. Samples from each time point were kept in temporal order, without clustering between columns, in order to preserve the circadian cycle sequence.

### 4.3 Antifungal Activity of the EOs

The phytopathogens *Botrytis cinerea* and *Colletotrichum gloeosporioides* were obtained from naturally infected plant tissues collected from plants exhibiting symptoms characteristic of gray mold and anthracnose, respectively, in the municipality of Barbacena, Minas Gerais. Fungal isolation was performed using the indirect method, which is widely employed in phytopathological studies because it enables the recovery of pure cultures from infected tissues. Initially, the plant material was washed in running water with neutral detergent to remove surface impurities and subsequently subjected to a surface sterilization process. Fragments measuring approximately 5 mm were excised from the transition zone between the lesioned tissue and the apparently healthy tissue, an area considered to have the highest pathogen activity due to the progression of the infection. These fragments were immersed in 70% ethyl alcohol for 30 s, followed by 0.1% sodium hypochlorite for 1 min, and then washed three times in sterile distilled water to remove residues of the sterilizing agents. Excess moisture was subsequently removed using sterile filter paper. All steps were carried out inside a sterile laminar flow hood.

Following sterilization, the fragments were aseptically transferred to Petri dishes containing approximately 15 mL of 2% Water‐Agar (WA) medium, using forceps that had been previously sterilized within the laminar flow hood. The plates were incubated in a BOD‐type growth chamber at 25 ± 2°C with a 12‐hour photoperiod for seven days, allowing for the initial development of fungal mycelium from the plant tissues. The use of WA medium, due to its low nutrient availability, favors the emergence of the pathogen directly from plant fragments and reduces the overgrowth of contaminants, facilitating the acquisition of monosporic cultures.

After colony development, the isolates were purified by transferring disks approximately 5 mm in diameter to plates containing Potato Dextrose Agar (PDA), a culture medium widely used for cultivating phytopathogenic fungi due to its high capacity to promote vigorous mycelial growth and abundant sporulation. The cultures were incubated again under the same conditions until pure, homogeneous, and contamination‐free colonies were obtained.

Isolate identification was based on macro and micromorphological characteristics described in specialized literature. For *Colletotrichum gloeosporioides*, characteristics such as colony color and appearance, the presence of acervuli, and the formation of conidial masses were evaluated, alongside conidial morphology (cylindrical to ellipsoidal shape with rounded, hyaline ends) (Figure 6) [44, 45, 46].

**FIGURE 6:**
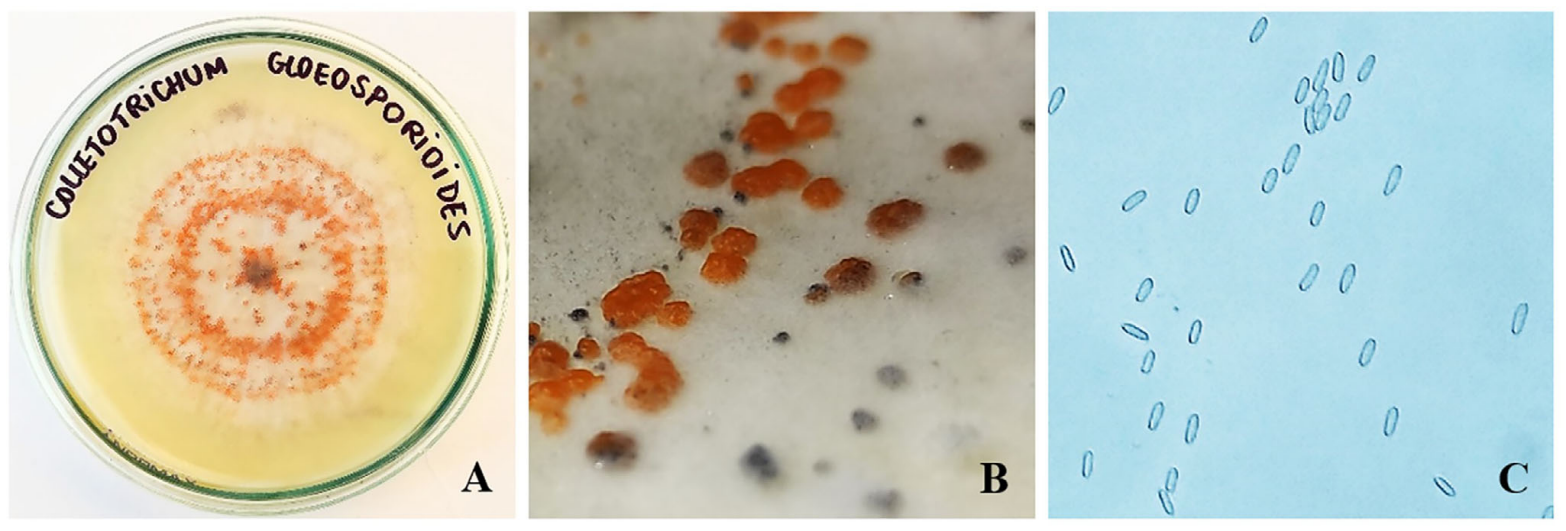
Morphological characteristics of *Colletotrichum gloeosporioides*: (A) culture image; (B) conidiomata; (C) conidia.

For *Botrytis cinerea*, identification was based on observing grayish mycelial growth, abundant production of branched conidiophores, and unicellular conidia (hyaline to slightly pigmented, oval to ellipsoidal), as well as the formation of the typical powdery appearance resulting from intense sporulation. Microscopic observations were performed using an optical microscope (Figure 7), and morphological characteristics were compared with taxonomic descriptions found in the literature to confirm the identity of the phytopathogens [47, 48].

**FIGURE 7:**
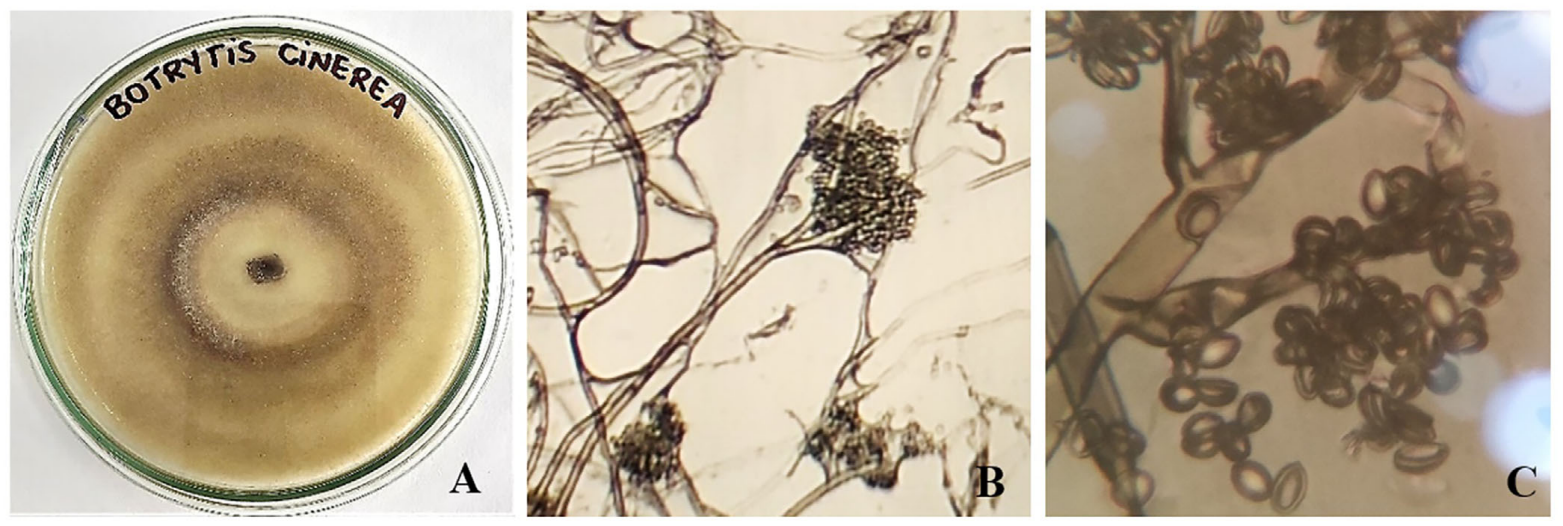
Morphological characteristics of *Botrytis cinerea*: (A) colony morphology on potato dextrose agar; (B) conidiophore; (C) conidia.

The antifungal activity of the EO was evaluated separately against the phytopathogens *Botrytis cinerea* and *Colletotrichum gloeosporioides*. For the biological assays, the EO obtained from *Callistemon viminalis* flowers, collected at 6:00 p.m. from collection site 2 (selected based on its chemical profile), was used.

After preparing and sterilizing the PDA culture medium, and after it was cooled and solidified, the EO was added at different concentrations. After homogenizing the EO in the culture medium, a 5 mm disc of the phytopathogen colony was added to the center of the Petri dish. The established concentrations were defined based on information from the literature on the use of EO in microorganisms. Thus, concentrations of 62.5, 125, 250 and 500 µL.mL^−1^ of the EO of *C. viminalis* flowers were tested using an aqueous solution of Tween 80 as a surfactant [49]. The antifungal Tebuconazole was used as a positive control at a concentration of 1 µL.mL^−1^, and the influence of a Tween 80 solution (5%) diluted in distilled water on the growth of the phytopathogen, which was used as a negative control, was also verified. The plates were kept in a BOD incubator at a temperature of 28°C. The experiment was conducted using a Completely Randomized Design (CRD). The plates were evaluated every 24 h after inoculation, measuring the colony diameter with a caliper. Evaluations were completed when the first colony reached the sides of the Petri dish. The values obtained for the colony diameter were subjected to Equation 2 to verify the PICM.

(2) $$\begin{array}{ccc} & & \mathrm{PICM}\left(\%\right)\\ & & =\frac{negative\;control\;growth\;-\;treatment\;growth}{negative\;control\;growth\;}\;\times \;100 \end{array}$$

The data were subjected to analysis of variance (ANOVA) using the SISVAR computer program [42], and the comparison between means was performed using Tukey's test at a 5% probability level.

## Author Contributions

**Chrystiaine Helena Campos de Matos**: data curation, investigation, writing – original draft. **Paloma Carolina de Lima Carvalho**: methodology, data curation. **João Paulo de Sousa**: methodology, data curation. **Luís Augusto Calsavara Costa**: methodology, data curation. **Jesus David Sierra Martinez**: formal analysis. **Alex Oliveira Botelho**: methodology. **Moacir Rossi Forim**: formal analysis. **Gracielle Oliveira Sabbag Cunha**: writing – review and editing. **Andreia Pereira Matos**: writing – review and editing. **Cássia Cristina Fernandes Alves**: conceptualization, methodology, supervision. **Cristiane de Melo Cazal**: conceptualization, methodology, supervision, writing – review and editing.

## Funding

This work was supported by the Fundação de Amparo à Pesquisa do Estado de Goiás (FAPEG) and the Coordenação de Aperfeiçoamento de Pessoal de Nível Superior (CAPES; project number 88887.616725/2021‐00).

## Conflicts of Interest

The authors declare no conflicts of interest.

## Data Availability

The data that support the findings of this study are available from the corresponding author upon reasonable request.

## References

[1] F. R. Saber , P. E. S. Munekata , K. Rizwan , et al., “Family Myrtaceae: The Treasure Hidden in the Complex/Diverse Composition,” Critical Reviews in Food Science and Nutrition 64 (2024): 6737–6755. 10.1080/10408398.2023.2173720 36748791

[2] H. E. Elsayed , I. M. Ayoub , M. S. Mady , and F. A. Moharram , “Chemical and Biological Characterization of *Melaleuca subulata* (Cheel) Craven Leaves' Volatile Constituents Supported by Chemometric Analysis and Molecular Docking,” BMC Complementary Medicine and Therapies 24 (2024): 76. 10.1186/s12906-024-04345-0 PMC10840179 38317130

[3] X. Liu , S. Wang , L. Cui , et al., “Flowers: Precious Food and Medicine Resources,” Food Science and Human Wellness 12 (2023): 1020–1052.

[4] H. S. Elshafie , A. Crescenzi , and I. Camele , “Investigating the Biological Effects of Plant Essential Oils on Plant‐Decaying Pathogens,” Plants 15 (2026): 542. 10.3390/plants15040542 PMC12944247 41754249

[5] D. C. Carrascal‐Hernández , E. Flórez‐López , Y. Peralta‐Ruiz , C. Chaves‐López , and C. D. Grande‐Tovar , “Eco‐Friendly Biocontrol Strategies of *Alternaria* Phytopathogen Fungus: A Focus on Gene‐Editing Techniques,” Agriculture 12 (2022): 1722.

[6] X. Li , T. Li , M. Li , et al., “Effect of Pathogenic Fungal Infestation on the Berry Quality and Volatile Organic Compounds of Cabernet Sauvignon and Petit Manseng Grapes,” Frontiers in Plant Science 13 (2022): 942487. 10.3389/fpls.2022.942487 PMC9353940 35937365

[7] H. Haghbayan , R. Moghimi , Y. Sarrafi , A. Taleghani , and R. Hosseinzadeh , “Enhancing Bioactivity of *Callistemon citrinus* (Curtis) Essential Oil Through Novel Nanoemulsion Formulation,” Journal of Biomaterials Science, Polymer Edition 35 (2024): 2660–2681. 10.1080/09205063.2024.2386787 39102358

[8] K. A. Veryer , B. Saygideğer , Ö. Süfer , Y. Saygideğer , A. Umay , and F. Bozok , “Biological Activities of *Callistemon citrinus* and *Callistemon viminalis* Essential Oils: EMT‐Linked Antitumor, Antioxidant, and Antibacterial Effects,” Chemistry & Biodiversity 23 (2026): e02563. 10.1002/cbdv.202502563 PMC13420218 41518656

[9] X. Zhang , X. Dai , T. Xiong , et al., “Essential Oil Extracted From *Callistemon viminalis* via Salting‐Out Assisted Hydrodistillation Enhances In Vitro Antioxidant and Antifungal Properties,” Industrial Crops and Products 232 (2025): 121248.

[10] T. A. Ankitha , N. U. Visakh , B. Pathrose , N. Mori , R. S. Baeshen , and R. Shawer , “Phytochemical Characterization of *Callistemon lanceolatus* Leaf Essential Oils and Their Application as Sustainable Stored Grain Protectants Against Major Storage Insect Pests,” Sustainability 16 (2024): 1055.

[11] M. Z. M. Salem , M. El‐Hefny , R. A. Nasser , H. M. Ali , N. A. El‐Shanhorey , and H. O. Elansary , “Medicinal and Biological Values of *Callistemon viminalis* Extracts: History, Current Situation and Prospects,” Asian Pacific Journal of Tropical Medicine 10 (2017): 229–237. 10.1016/j.apjtm.2017.03.015 28442106

[12] L. Anjos da Silva , R. Santos da Silva , M. Rodrigues de Oliveira , A. C. Guimarães , and R. Takeara , “Chemical Composition and Biological Activities of Essential Oils From Myrtaceae Species Growing in Amazon: An Updated Review,” Journal of Essential Oil Research 28 (2023): 1–14.

[13] T. Kabdal , R. Kumar , O. Prakash , et al., “Seasonal Variation in the Essential Oil Composition and Biological Activities of *Thymus linearis* Benth. Collected From the Kumaun Region of Uttarakhand, India,” Biochemical Systematics and Ecology 103 (2022): 104449.

[14] C. H. C. Matos , P. C. L. Carvalho , A. O. Botelho , et al., “Influence of Extraction Time and Circadian Rhythm on the Content, Chemical Composition and Antifungal Activity of the Essential Oils from *Callistemon viminalis* (Myrtaceae) Leaves,” Journal of the Brazilian Chemical Society 36 (2025): e20250021.

[15] C. H. Pires , J. A. M. Paula , L. M. F. Tresvenzol , P. H. Ferri , T. S. Fiuza , and M. T. F. Bara , “Composição Química e Atividade Antimicrobiana Dos Óleos Essenciais das Folhas e Flores de *Callistemon viminalis* (Sol. ex Gaertn.) G. Don ex Loudon (Myrtaceae),” Revista de Ciências Farmacêuticas Básica e Aplicada 34 (2013): 597–601.

[16] R. A. Larayetan , O. O. Okoh , A. Sadimenko , and A. I. Okoh , “Terpene Constituents of the Aerial Parts, Phenolic Content, Antibacterial Potential, Free Radical Scavenging and Antioxidant Activity of *Callistemon citrinus* (Curtis) Skeels (Myrtaceae) from Eastern Cape Province of South Africa,” BMC Complementary and Alternative Medicine 17 (2017): 292. 10.1186/s12906-017-1804-2 PMC5460465 28583128

[17] D. Kumar , M. Sukapaka , G. D. K. Babu , and Y. Padwad , “Chemical Composition and In Vitro Cytotoxicity of Essential Oils From Leaves and Flowers of *Callistemon citrinus* From Western Himalayas,” PLoS ONE 10 (2015): e0133823. 10.1371/journal.pone.0133823 PMC4550473 26308916

[18] L. N. Misra , F. Huq , A. Ahmad , and A. K. Dixit , “Chemical Composition of the Essential Oils of *Callistemon lanceolatus* DC and *C. polandii* F.M. Bailey,” Journal of Essential Oil Research 9 (1997): 625–628.

[19] A. M. Kamal , M. I. S. Abdelhady , N. F. Tawfik , and S. M. Abdelkhalik , “Lipoidal Matter and Essential Oil Contents of Leaves and Fowers of *Callistemon viridiflorus* and Their Antimicrobial Activity,” Journal of Essential Oil Bearing Plants 16 (2013): 771–780.

[20] Instituto Nacional de Metereologia (INMET), accessed May, 2026, https://tempo.inmet.gov.br/TabelaEstacoes/A001.

[21] P. V. L. Santos , E. D. N. S. da Cruz , J. D. A. Nunes , et al., “Seasonal Influence on Volatile Composition of *Psidium friedrichsthalianum* Leaves, Sampled in the Brazilian Amazon,” Horticulturae 9 (2023): 768.

[22] G. Crestani , K. Večeřová , N. Cunningham , U. O. Badmus , O. Urban , and M. A. K. Jansen , “Comprehensive Modulation of Secondary Metabolites in Terpenoid‐Accumulating *Mentha spicata* L. Via UV Radiation,” Plants 13 (2024): 1746. 10.3390/plants13131746 PMC11243551 38999586

[23] H. A. Gad , I. M. Ayoub , and M. Wink , “Phytochemical Profiling and Seasonal Variation of Essential Oils of Three *Callistemon* Species Cultivated in Egypt,” PLoS ONE 14 (2019): e0219571. 10.1371/journal.pone.0219571 PMC6622538 31295290

[24] R. Gogoi , T. Begum , N. Sarma , S. K. Pandey , and M. Lal , “Chemical Composition of *Callistemon Citrinus* (Curtis) Skeels Aerial Part Essential Oil and Its Pharmacological Applications, Neurodegenerative Inhibitory, and Genotoxic Efficiencies,” Journal of Food Biochemistry 45 (2021): e13767. 10.1111/jfbc.13767 34028046

[25] O. Rabie , H. A. S. El‐Nashar , T. A. Majrashi , et al., “Chemical Composition, Seasonal Variation and Antiaging Activities of Essential Oil From *Callistemon subulatus* Leaves Growing in Egypt,” Journal of Enzyme Inhibition and Medicinal Chemistry 38 (2023): 2224944. 10.1080/14756366.2023.2224944 PMC10304439 37369580

[26] I. J. M. M. Silva , C. C. Fernandes , N. B. S. Silva , et al., “Chemical Composition and Antimycobacterial, Anti‐ *Malassezia furfur* and Cytotoxic Activities of Essential Oils From *Callistemon viminalis* (Sol. ex Gaertn.) G. Don Leaves and Flowers,” Natural Product Research 39 (2024): 5352–5356. 10.1080/14786419.2024.2386120 39078872

[27] R. P. Adams , Identification of Essential Oil Components by Gas Chromatography/Mass Spectrometry, 4th ed. (Allured Pub Corp, 2017).

[28] Z. Hashemifar , F. Sanjarian , H. Naghdi Badi , and A. Mehrafarin , “Impact of Varying Light Intensities on Morphology, Phytochemistry, Volatile Compounds, and Gene Expression in *Thymus vulgaris* L,” PLoS ONE 20 (2025): e0317840. 10.1371/journal.pone.0317840 PMC11864514 40009615

[29] X. Yu , X. Qi , S. Li , et al., “Transcriptome Analysis of Light‐Regulated Monoterpenes Biosynthesis in Leaves of *Mentha canadensis* L,” Plants 10 (2021): 930. 10.3390/plants10050930 PMC8148558 34066919

[30] L. Frank , M. Wenig , A. Ghirardo , et al., “Isoprene and β‐Caryophyllene Confer Plant Resistance via Different Plant Internal Signalling Pathways,” Plant, Cell & Environment 44 (2021): 1151–1164. 10.1111/pce.14010 33522606

[31] Y. C. Chuang , M. C. Lee , Y. L. Chang , W. H. Chen , and H. H. Chen , “Diurnal Regulation of the Floral Scent Emission by Light and Circadian Rhythm in the Phalaenopsis Orchids,” Botanical Studies 58 (2017): 50. 10.1186/s40529-017-0204-8 PMC5688052 29143225

[32] O. Basallo , A. Lucido , A. Sorribas , et al., “Modeling the Effect of Daytime Duration on the Biosynthesis of Terpenoid Precursors,” Frontiers in Plant Science 15 (2024): 1465030. 10.3389/fpls.2024.1465030 PMC11609946 39624244

[33] J. Baptiste Hzounda Fokou , P. Michel Jazet Dongmo , and F. Fekam Boyom , “Essential Oil's Chemical Composition and Pharmacological Properties,” in Essential Oils—Oils of Nature (IntechOpen, 2020).

[34] D. M. Pilatti , A. M. T. Fortes , T. C. M. Jorge , and N. P. Boiago , “Comparison of the Phytochemical Profiles of Five Native Plant Species in Two Different Forest Formations,” Brazilian Journal of Biology 79 (2019): 233–242. 10.1590/1519-6984.179526 29924133

[35] S. Kumari , S. Pundhir , P. Priya , et al., “EssOilDB: A Database of Essential Oils Reflecting Terpene Composition and Variability in the Plant Kingdom,” Database 2014 (2014): 1–12. 10.1093/database/bau120 PMC4273207 25534749

[36] M. E. I. Badawy and S. A. M. Abdelgaleil , “Composition and Antimicrobial Activity of Essential Oils Isolated From Egyptian Plants Against Plant Pathogenic Bacteria and Fungi,” Industrial Crops and Products 52 (2014): 776–782.

[37] K. Okazaki , H. Sumitani , K. Takahashi , and Y. Isegawa , “Mode of Antifungal Action of Daito‐Gettou (*Alpinia zerumbet* var. exelsa) Essential Oil Against *Aspergillus brasiliensis* ,” Foods 12 (2023): 1298. 10.3390/foods12061298 PMC10048414 36981224

[38] Z. Shahina , R. Al Homsi , J. D. W. Price , M. Whiteway , T. Sultana , and T. E. S. Dahms , “Rosemary Essential Oil and Its Components 1,8‐Cineole and α‐Pinene Induce ROS‐Dependent Lethality and ROS‐Independent Virulence Inhibition in *Candida albicans* ,” PLoS ONE 17 (2022): e0277097. 10.1371/journal.pone.0277097 PMC9668159 36383525

[39] D. Yu , J. Wang , X. Shao , F. Xu , and H. Wang , “Antifungal Modes of Action of Tea Tree Oil and Its Two Characteristic Components Against *Botrytis cinerea* ,” Journal of Applied Microbiology 119 (2015): 1253–1262. 10.1111/jam.12939 26294100

[40] K. Połeć , A. Wójcik , M. Flasiński , P. Wydro , M. Broniatowski , and K. Hąc‐Wydro , “The Influence of Terpinen‐4‐ol and Eucalyptol ‐ The Essential Oil Components ‐ on Fungi and Plant Sterol Monolayers,” Biochimica et Biophysica Acta (BBA): Biomembranes 1861 (2019): 1093–1102. 10.1016/j.bbamem.2019.03.015 30926362

[41] D. Bomfim de Barros , L. de Oliveira E Lima , L. Alves da Silva , et al., “α‐Pinene: Docking Study, Cytotoxicity, Mechanism of Action, and Anti‐Biofilm Effect Against *Candida albicans* ,” Antibiotics 12 (2023): 480. 10.3390/antibiotics12030480 PMC10044001 36978347

[42] D. F. Ferreira , “SISVAR: A Computer Statistical Analysis System,” Ciência e Agrotecnologia 35 (2011): 1039–1042.

[43] Ø. Hammer , D. A. T. Harper , and P. D. Ryan , “Past: Paleontological Statistics Software Package for Education and Data Analysis,” Palaeontologia Electronica 4 (2001): 1–9.

[44] S. Barhoom and A. Sharon , “cAMP Regulation of “Pathogenic” and “Saprophytic” Fungal Spore Germination,” Fungal Genetics and Biology 41 (2004): 317–326. 10.1016/j.fgb.2003.11.011 14761792

[45] S. Alaniz , V. Cuozzo , V. Martínez , M. J. Stadnik , and P. Mondino , “Ascospore Infection and *Colletotrichum* Species Causing Glomerella Leaf Spot of Apple in Uruguay,” The Plant Pathology Journal 35 (2019): 100–111. 10.5423/PPJ.OA.07.2018.0145 PMC6464197 31007640

[46] Q.‐H. Wang , X.‐Y. Li , T.‐T. Xu , et al., “Identification and Characterization of *Colletotrichum gloeosporioides* Sensu Stricto, the Agent of Anthracnose Disease of *Sorbaria sorbifolia* in China,” Journal of General Plant Pathology 87 (2021): 71–76.

[47] C. Krasnow and C. Ziv , “Non‐Chemical Approaches to Control Postharvest Gray Mold Disease in Bell Peppers,” Agronomy 12 (2022): 216.

[48] G. Holz , S. Coertze , and B. Williamson , “The Ecology of *Botrytis* on Plant Surfaces,” in Botrytis: Biology, Pathology and Control (Springer, 2007), 9–27.

[49] A. C. A. Santos , M. Rossato , L. A. Serafini , et al., “Efeito Fungicida dos Óleos Essenciais De *Schinus molle* L. e *Schinus terebinthifolius* Raddi, Anacardiaceae, Do Rio Grande Do Sul,” Revista Brasileira de Farmacognosia 20 (2010): 154–159.

